# How alkalinization drives fungal pathogenicity

**DOI:** 10.1371/journal.ppat.1006621

**Published:** 2017-11-09

**Authors:** Tânia R. Fernandes, David Segorbe, Dov Prusky, Antonio Di Pietro

**Affiliations:** 1 Departamento de Genética, Universidad de Córdoba, Córdoba, Spain; 2 Department of Postharvest Science of Fresh Produce, A.R.O., Bet Dagan, Israel; THE SAINSBURY LABORATORY, UNITED KINGDOM

## Introduction

pH governs most, if not all, processes of life. In fungi, ambient pH acts as a potent regulator of growth and development [1]. Studies conducted primarily in the 2 model organisms *Saccharomyces cerevisiae* and *Aspergillus nidulans* have cemented our understanding of how fungi sense and respond to pH. More recently, pH has emerged as a key player in the control of fungal pathogenicity. Infections caused by fungi are often associated with a pH shift in the surrounding host tissue [2–4]. Extracellular alkalinization contributes to fungal virulence, but the underlying mechanisms are not fully understood. Recent studies have revealed new and unexpected ways by which fungi induce host alkalinization to increase their infectious potential. Here, we provide a brief overview of the mechanisms that govern pH signaling in fungi and highlight how recent findings have advanced our understanding of pathogen-induced alkalinization and its role during infection. We also discuss the emerging view that intracellular pH (pHi) acts as a master switch to govern fungal development and pathogenicity.

## Alkaline pH sensing and adaptation

High pH imposes severe stress on the fungal cell, including difficulties in the acquisition of nutrients or reduced availability of essential elements, such as iron or copper [5]. Fungi respond to alkaline pH through the dedicated Pal/Rim signaling pathway, which is widely conserved among ascomycetes and basidiomycetes and essential for growth at high pH [1, 6]. Signaling upon a shift to alkaline pH is initiated by the 7-transmembrane domain receptor PalH/Rim21. How exactly PalH/Rim21 senses changes in pH is not fully understood. Recent work in *S*. *cerevisiae* suggests that the C-terminal cytosolic domain detects altered lipid asymmetry of the plasma membrane as a result of alkaline-induced depolarization [7]. At high pH, PalH/Rim21 mediates ubiquitination and phosphorylation of its interaction partner, the α-arrestin PalF/Rim8, resulting in endocytosis of the receptor complex and recruitment of the endosomal sorting complexes required for transport (ESCRT) scaffold to plasma membrane-associated foci [1]. Ultimately, this leads to processing and activation of the zinc finger transcription factor PacC/Rim101. In *A*. *nidulans*, PacC is cleaved from the full-length 72-kDa to the 27-kDa active form by 2 successive C-terminal proteolytic cleavages, the first of which is carried out by the signaling protease PalB. Processed PacC protein functions both as an activator of alkaline-expressed genes and a repressor of acidic-expressed genes, thereby orchestrating the cellular response to alkaline pH [6].

Ambient pH adaptation ensures the expression of the adequate set of genes at a given pH. This is crucial during fungal infection to ensure, for example, the correct deployment of virulence factors that function at a specific pH [2, 8, 9]. The Pal/Rim pathway was shown to be essential for infection in a number of fungal pathogens of humans, such as *Candida albicans*, *Fusarium oxysporum*, and *A*. *fumigatu*s [8, 10, 11]. In plant pathogenic fungi, PacC contributes to virulence in necrotrophic or postharvest pathogens [12–14] but is dispensable in others, such as the hemibiotrophic root-infecting fungus *F*. *oxysporum* [15]. These results reveal contrasting roles of PacC, most likely associated with distinct modes of host infection of the different phytopathogens.

## Alkalinization mechanisms

Fungal pathogens have been known for decades to adjust the extracellular pH in order to increase their infectious potential [3, 16]. Alkalinization of the plant host was first reported in a number of fruit-infecting species, such as *Colletotrichum* spp. and *Alternaria alternata* [3, 17], and more recently in the root-infecting fungus *F*. *oxysporum* [18]. These fungi are able to trigger an increase of more than 2 units in the pH of the surrounding fruit tissue or the rhizosphere, respectively. Similarly, the human pathogen *C*. *albicans* raises the pH in host macrophages by several units, resulting in neutralization of the normally acidic phagosome [19, 20].

The main mechanism of host alkalinization reported in these fungal species is the release of ammonia, which acts as a weak base [17, 19]. Concentrations of up to 5 mM ammonia have been measured in colonized fruit tissue [21]. The exact mechanism that leads to extracellular accumulation of ammonia remains to be elucidated. Work in *S*. *cerevisiae*, *C*. *albicans*, and *Colletotrichum gloeosporioides* showed that this process requires the regulated uptake of amino acids via amino acid permeases or their mobilization from vacuolar stores, followed by catabolism through different routes involving steps of deamination [19, 22, 23]. In *C*. *gloeosporioides*, the transformation of glutamate to α-ketoglutarate and ammonium was shown to be carried out by the NAD^+^-specific glutamate dehydrogenase Gdh2 [22]. A second requirement for ammonia-mediated alkalinization is carbon deprivation. Presumably, a lack of carbon prevents the efficient use of ammonia for biosynthesis of amino acids and nucleotides, favoring its accumulation [19, 23]. To protect the cell from the toxic effects, ammonia is released either by passive diffusion or through the action of transporters, such as the members of the Ato protein family [19, 24, 25]. The precise mechanisms of ammonia extrusion during alkalinization remain to be determined.

Phytopathogens have been traditionally classified into acidifiers and alkalinizers based on their strategy to either decrease or increase the pH of the surrounding host tissue during infection [3]. However, this distinction might be less clear-cut than previously assumed. A recent study involving different fruit-infecting fungi revealed that each of them could induce either alkalinization or acidification of the environment, depending on the availability of carbon. Carbon limitation triggered extracellular accumulation of ammonia and alkalinization, whereas an excess of carbon induced acidification through the release of gluconic acid [23]. These findings are of biological relevance because pathogens are likely to encounter different levels of carbon availability, depending on the host niche or the stage of infection (biotrophic or necrotrophic). For example, a postharvest pathogen will be exposed to gradually increasing sugar levels as the fruit ripens and therefore may undergo a switch from alkalinization to acidification during the infection process.

**Fig 1:**
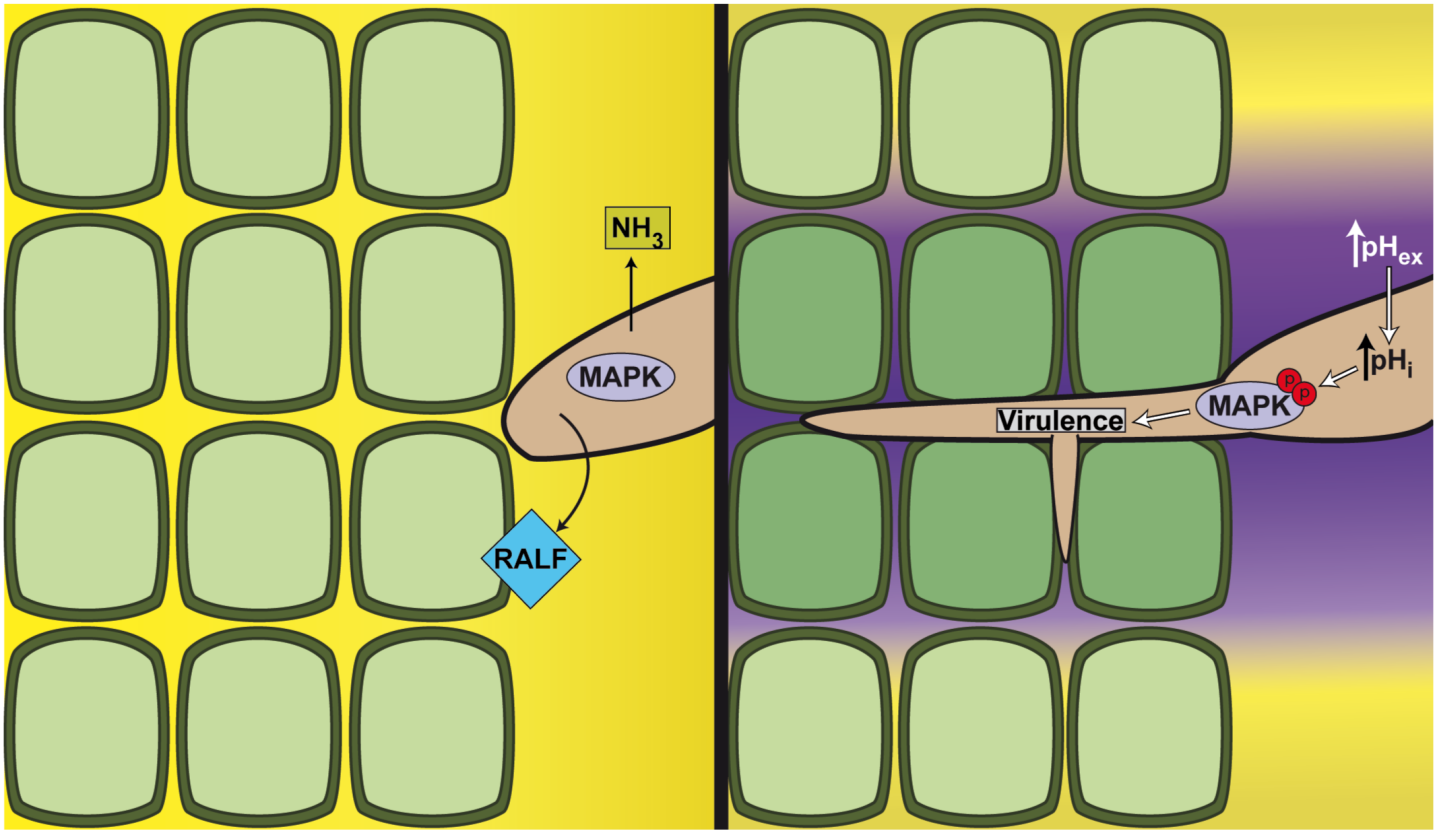
Host alkalinization drives virulence in fungal pathogens. During infection, fungal pathogens induce alkalinization of the surrounding host tissue through regulated release of ammonia and, in certain phytopathogens, by secreting small regulatory peptides that mimic plant RALFs (left panel). The resulting increase in extracellular pH activates the fungal IG MAPK cascade, likely via modulation of pHi, to trigger phosphorylation of the IG MAPK and morphogenetic transition towards infectious growth (right panel). Yellow color denotes acidic pH, while purple denotes neutral to alkaline pH. IG, invasive growth; MAPK, mitogen-activated protein kinase; pHi, intracellular pH; RALF, Rapid ALkalinizing Factor.

To efficiently alkalinize the plant tissue through the release of ammonia, fungal pathogens must first build up a significant amount of hyphal biomass. How, then, is alkalinization achieved during early stages of infection when only a low number of hyphae are present in the host? To overcome this limitation, some biotrophic and hemibiotrophic pathogens have co-opted a pH regulatory mechanism that is naturally present in the plant host. *F*. *oxysporum* was recently shown to secrete a functional homologue of Rapid ALkalinizing Factor (RALF), a family of conserved plant regulatory peptides [26]. Similar to plant RALFs, the *Fusarium* RALF (F-RALF) peptide triggers rapid alkalinization of the apoplast [18]. Isogenic *F*. *oxysporum* mutants lacking functional F-RALF failed to induce root alkalinization and showed markedly reduced virulence in tomato plants. Intriguingly, these strains also provoked a strong host immune response. F-RALF appears to target the plant receptor-like kinase FERONIA, which also mediates responses to endogenous plant RALF peptides. An *Arabidopsis* mutant defective in FERONIA failed to respond to fungal F-RALF and displayed enhanced resistance against *F*. *oxysporum* [18]. While the details on the mode of action of F-RALF remain to be elucidated, it was recently shown that endogenous RALF–FERONIA signaling leads to inactivation of the plasma membrane H^+^-ATPase AHA2 and inhibition of plant cell elongation [27]. Moreover, FERONIA integrates signals from other plant hormones, such as auxin and abscisic acid [28]. Intriguingly, fungal RALF homologues are found in phylogenetically distant species, spanning both ascomycetes and basidiomycetes as well as hemibiotrophs and biotrophs [18, 29]. This taxonomically discontinuous distribution suggests that co-option of FERONIA by fungal RALF peptides was acquired multiple times during evolution. In summary, these studies illustrate how fungal pathogens have evolved multiple ways to manipulate host pH during different stages of infection (Fig 1).

## How alkalinization controls pathogenicity

One of the key questions is how an increase in host pH induced by the pathogen promotes infection. Different mechanisms have been proposed, based either on the host or the fungal cell machinery. For example, ammonium secretion by *Colletotrichum coccodes* was shown to activate plant NADPH oxidases and to enhance host cell death [30]. On the other hand, alkaline pH triggers PacC/Rim101-mediated expression of fungal genes encoding virulence factors, such as the cell wall–degrading enzymes pectate lyase and endoglucanase from *C*. *gloeosporioides*, which display maximum activity at alkaline pH typically observed in decaying fruit tissue [2]. *C*. *albicans PHR1*, a gene encoding a cell wall remodeling β(1,3)-glucanosyltransferase, which is fundamental for host tissue adhesion and invasion, is up-regulated at alkaline pH via Rim101 [31].

Alkalinization has also been associated with infection-related morphogenetic changes. Ammonia release during germination of *C*. *gloeosporioides* conidia led to enhanced formation of specialized infection structures called apressoria [21]. In *C*. *albicans*, an upshift in pH promotes the transition from the unicellular yeast to the filamentous hyphal form [4, 8, 19]. This morphogenetic switch, which is critical for virulence in mammalian hosts, is mediated by a number of cell signaling pathways, including the Pal/Rim route and the invasive growth (IG) mitogen-activated protein kinase (MAPK) cascade [8, 32].

The IG MAPK pathway is broadly conserved in fungi and essential for infection in a wide range of plant pathogens [33]. A recent study in *F*. *oxysporum* revealed that extracellular alkalinization triggers rapid phosphorylation of the IG MAPK, leading to enhanced IG and virulence in tomato plants (Fig 1) [18]. Collectively, these studies show that alkalinization promotes infection-related fungal development and morphogenesis through conserved signaling pathways, such as the Pal/Rim or the IG MAPK cascade, although the cellular mechanisms linking the pH shift to MAPK activity remain to be elucidated.

## pHi: A master switch for pathogenicity?

In contrast to ambient pH, pHi tends to be constant and tightly regulated [34]. Nevertheless, rapid changes in pHi can occur in response to different stimuli, such as shifts in extracellular pH or nutrient status [35]. For example, a rapid and transitory decrease of pHi upon extracellular acidification was detected in an *Aspergillus niger* strain, expressing the pH-sensitive green fluorescent protein (GFP) variant pHluorin [36]. It is increasingly appreciated that pHi acts as a general regulator of cellular functions, such as growth and proliferation [37], life span [38], and nutrient response [39]. So far, the role of pHi in fungal infection has not been examined in detail, but it is conceivable that it could act as a signal linking extracellular alkalinization to activation of the IG MAPK and pathogenicity (Fig 1).

Two major mechanisms for pHi regulation have been reported in fungi, both based on conserved proton-pumping ATPases. The primary determinant of cytosolic pH is Pma1, an essential H^+^-ATPase and the most abundant plasma membrane protein in *S*. *cerevisiae*. Pma1 homologues are found in all fungi, as well as in plants. The second mechanism is the vacuolar ATPase (V-ATPase), a multiprotein complex that mediates acidification of organelles, such as vacuoles, endosomes, or the Golgi [34]. Pma1 and V-ATPase are often coregulated, for example in response to sudden shifts in ambient pH or glucose levels. In *S*. *cerevisiae*, high glucose levels lead to the activation of Pma1 via phosphorylation of conserved C-terminal residues and also promote assembly of the V-ATPase complex. By contrast, glucose depletion results in Pma1 autoinhibition and disassembly of the V-ATPase complex, concomitant with an acidification of the cytosol [34, 39]. As a general rule, activation of these proton-pumping ATPases leads to a pHi increase due to increased proton export, whereas their inhibition triggers intracellular acidification.

Meanwhile, a link between H^+^-ATPases and virulence is beginning to emerge. In the 2 dimorphic fungi *Histoplasma capsulatum* and *C*. *albicans*, mutants defective in V-ATPase were blocked in the yeast-hypha transition and showed attenuated virulence in a murine infection model [40, 41]. Likewise, a strain of the rice blast fungus *Magnaporthe oryzae* lacking a subunit of the V-ATPase complex exhibited reduced vacuolar acidification and produced small, nonfunctional apressoria. Interestingly, the mutant was unable to cause disease even when inoculated through wounds, suggesting that V-ATPase is not only required for infection-related morphogenesis but also for additional pathogenicity functions [42]. Recently, a paralog of Pma1 named Pma2 was identified during 2 independent genetic screens for pathogenicity genes in *M*. *oryzae* and *Colletotrichum higginsianum* [43, 44]. In contrast to *pma1*, which is constitutively expressed, *pma2* was up-regulated specifically during apressoria formation and infection [43, 44]. A deletion mutant of *C*. *higginsianum* lacking *pma2* failed to penetrate the host plant, consistent with a crucial role of this H^+^-ATPase in fungal virulence [43]. In *M*. *oryzae*, *pma2* expression was up-regulated by ammonia [45], suggesting that *pma2* expression during infection might be activated by pathogen-induced alkalinization. Pma2 is structurally related to its broadly conserved paralog Pma1 but exhibits some key differences, such as the lack of conserved phosphorylation sites at the C-terminus, which could be related to the differential regulation during infection. Interestingly, the Pma2 clade is present in many phytopathogenic ascomycetes and absent in nonpathogenic species. Thus, the acquisition of a second plasma membrane H^+^-ATPase, in addition to Pma1, with a specific role in virulence, appears to represent a common strategy of ascomycete pathogens. Enhanced activity of Pma2 and V-ATPase during infection, with the concomitant rise of pHi, could trigger conserved components of the pathogenicity signaling machinery, such as the IG MAPK cascade.

## Future challenges

While our understanding of the role of alkalinization in fungus–host interaction has advanced considerably, important questions remain. For example, the precise mechanism mediating the release of ammonia from the fungal cell remains to be established. Further research is also needed to elucidate the pH-sensing processes that link extracellular alkalinization to known pathogenicity signaling modules, such as the Pal/Rim pathway or the IG MAPK cascade. Besides the plasma membrane protein PalH, whose precise mode of pH sensing remains to be defined, additional pH sensors must exist. Some of these may be intracellular, in line with the emerging role of pHi as a master regulator of fungal development and virulence. The discovery of novel pH-sensing and regulatory components will likely require forward or reverse genetic screens in fungal model organisms, such as *S*. *cerevisiae* or *Neurospora crassa*, which could be based on conserved downstream readouts, including MAPK cascades. However, these approaches might be made difficult by the essential nature of some of the pH regulatory genes, as exemplified by the H^+^-ATPase Pma1. The exciting discovery that fungi highjack plant regulatory peptides to enhance alkalinization adds yet another example to the ongoing arms race between pathogen and host and may pave the way for further discoveries of cross-kingdom pH regulation. Finally, the recent finding that different antifungals induce a dramatic decrease of pHi in *Candida* [46, 47], combined with the broadly conserved role of alkalinization in virulence, makes alkalinization an attractive target for the control of fungal pathogens.
